# Pyruvate dehydrogenase kinase 1 protects against neuronal injury and memory loss in mouse models of diabetes

**DOI:** 10.1038/s41419-023-06249-2

**Published:** 2023-11-07

**Authors:** Yuan Yao, Jiaming Shi, Chunlai Zhang, Wei Gao, Ning Huang, Yaobei Liu, Weiwen Yan, Yingguang Han, Wenjuan Zhou, Liang Kong

**Affiliations:** 1 Department of Clinical Laboratory, Affiliated Hospital of Shandong University of Traditional Chinese Medicine, Jinan, Shandong China; 2 Key Laboratory of the Ministry of Education for Experimental Teratology, Shandong Provincial Key Laboratory of Mental Disorders, Department of Human Anatomy and Histoembryology, School of Basic Medical Sciences, Cheeloo College of Medicine, Shandong University, Jinan, Shandong China; 3 Department of Clinical Laboratory, Zibo Hospital of Traditional Chinese Medicine, Zibo, Shandong China

**Keywords:** Cell death in the nervous system, Neurodegeneration

## Abstract

Hyperglycemia-induced aberrant glucose metabolism is a causative factor of neurodegeneration and cognitive impairment in diabetes mellitus (DM) patients. The pyruvate dehydrogenase kinase (PDK)–lactic acid axis is regarded as a critical link between metabolic reprogramming and the pathogenic process of neurological disorders. However, its role in diabetic neuropathy remains unclear. Here, we found that PDK1 and phosphorylation of pyruvate dehydrogenase (PDH) were obviously increased in high glucose (HG)-stimulated primary neurons and Neuro-2a cell line. Acetyl-coA, a central metabolic intermediate, might enhance PDK1 expression via histone H3K9 acetylation modification in HG condition. The epigenetic regulation of PDK1 expression provided an available negative feedback pattern in response to HG environment-triggered mitochondrial metabolic overload. However, neuronal PDK1 was decreased in the hippocampus of streptozotocin (STZ)-induced diabetic mice. Our data showed that the expression of PDK1 also depended on the hypoxia-inducible factor-1 (HIF-1) transcriptional activation under the HG condition. However, HIF-1 was significantly reduced in the hippocampus of diabetic mice, which might explain the opposite expression of PDK1 in vivo. Importantly, overexpression of PDK1 reduced HG-induced reactive oxygen species (ROS) generation and neuronal apoptosis. Enhancing PDK1 expression in the hippocampus ameliorated STZ-induced cognitive impairment and neuronal degeneration in mice. Together, our study demonstrated that both acetyl-coA-induced histone acetylation and HIF-1 are necessary to direct PDK1 expression, and enhancing PDK1 may have a protective effect on cognitive recovery in diabetic mice.

**Figure:**
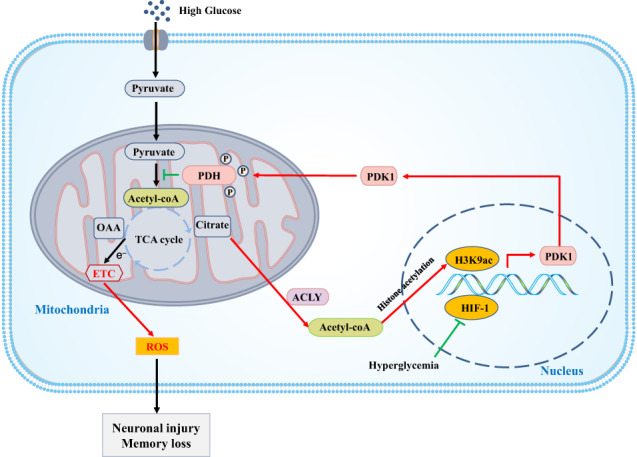
Schematic representation of the protective effect of PDK1 on hyperglycemia-induced neuronal injury and memory loss. High glucose enhanced the expression of PDK1 in an acetyl-coA-dependent histone acetylation modification to avoid mitochondrial metabolic overload and ROS release. However, the decrease of HIF-1 may impair the upregulation of PDK1 under hyperglycemia condition. Overexpression of PDK1 prevented hyperglycemia-induced hippocampal neuronal injury and memory loss in diabetic mice.

Schematic representation of the protective effect of PDK1 on hyperglycemia-induced neuronal injury and memory loss. High glucose enhanced the expression of PDK1 in an acetyl-coA-dependent histone acetylation modification to avoid mitochondrial metabolic overload and ROS release. However, the decrease of HIF-1 may impair the upregulation of PDK1 under hyperglycemia condition. Overexpression of PDK1 prevented hyperglycemia-induced hippocampal neuronal injury and memory loss in diabetic mice.

## Introduction

Hyperglycemia is a prevalent characteristic of diabetes mellitus (DM) patients [1]. Chronic hyperglycemia induces persistent glucotoxicity in the brain and leads to serious neuropathological changes, such as neurodegeneration, neuroinflammation and cognitive dysfunction [2–4]. The mechanisms of glucotoxicity mainly focused on increased oxidative stress, advanced glycation end products and excess production of intermediates-induced post-translational modification of proteins [2, 5]. All of these factors are closely associated with abnormal metabolism in the diabetic brain [6, 7]. In fact, excess pyruvate from glycolysis induces an overload of mitochondrial metabolism and increases the generation of reactive oxygen species (ROS) [8]. The accumulation of ROS impairs the activities of mitochondrial electron transfer chain components such as aconitate hydratase and complex I, and finally leads to mitochondrial dysfunction, inflammation, RNA and DNA damage, Aβ oxidation and lipid peroxidation [3, 9]. Therefore, it is of great significance to investigate a new therapeutic strategy to reprogram abnormal metabolism and decrease oxidative stress in hyperglycemia-induced neurological apoptosis and cognitive defects.

Growing evidence indicates that the pyruvate dehydrogenase kinase (PDK)‒lactic acid axis plays key roles in mitochondrial metabolic dysfunction and the pathophysiology of neurological diseases [10, 11]. PDKs (PDK1–4) are important regulator enzymes of mitochondrial aerobic metabolism, which can inhibit the activity of the pyruvate dehydrogenase (PDH) complex by phosphorylation of PDH and prevent pyruvate covert into acetyl-CoA. Three specific phosphorylation sites (Ser^232^, Ser^293^, and Ser^300^) of the E1 PDH component by PDKs are reported as previously described [10]. PDK2 and PDK4 have been largely investigated in diabetic neuropathy. In diabetic mice model, PDK2 and PDK4 have been proven to participate in the activation of satellite glial cells and pain hypersensitivity in the dorsal root ganglion, and regulation of inflammatory response and feeding behavior in the astrocyte of the hypothalamus [12, 13]. Unlike PDK2 and PDK4, PDK1 is highly expressed in neurons [14]. Research showed that Aβ-resistant nerve cells displayed increased PDK1 expression, enhanced lactate dehydrogenase A (LDHA) activity and reduced mitochondrial ROS, suggesting the potential anti-apoptotic roles of the PDK-lactic acid axis in the surviving neurons of the Alzheimer’s disease (AD) brain [15–17]. However, the effect of PDK1 on diabetic neuropathy of the central nervous system (CNS) has been ignored until now. The apoptotic-resistance mechanisms of the PDK1-lactic acid axis in Aβ-resistant nerve cells may give us new inspiration to find effective therapy for diabetic neuropathy.

The brain is a high energy demand organ and most of glucose is utilized to sustain neuronal activity and information processing in neurons. Health neurons have the self-protection ability to resist extreme circumstances, such as hypoxic and hypoglycemia to some extent [18–20]. Hypoxia-inducible factor-1 (HIF-1), as an essential regulator of metabolism, may have neuroprotective effects in cerebral ischemia/reperfusion injury and AD [15, 21, 22]. PDK1 has been reported to be an important HIF-1-target gene in hypoxic B lymphocyte cell lines [23]. Therefore, in this study, we aimed to investigate the role and mechanism of PDK1 in hyperglycemia-induced abnormal metabolism and neuronal injury.

## Materials and methods

### Animals

Male C57BL/6 mice aged 8–10 weeks were used in the following experiments. Animals were bred separately with enough food and water under a 12 h day/night cycle at a constant comfortable temperature of 22 ± 2 °C. Mice were randomly assigned to experimental groups.

### Streptozotocin-induced diabetes models

According to previous research, two mouse models of diabetes were built in this study [4, 12]. The first is a single intraperitoneal injection of a high dose of 220 mg/kg streptozotocin (STZ, Sigma-Aldrich, St. Louis, MO) in 0.1 M sodium citrate buffer (pH 4.4). Another mild type 1 diabetes model is induced by multiple injections of low doses of 40 mg/kg STZ (MLDS) for five consecutive days. Fasting blood glucose levels were measured 3 days after a single STZ injection and 7 days of MLDS injections using an FAD-GDH System (Sanocare, Changsha, China). Animals with blood glucose higher than 16.7 mM were used in the following experiment.

### Cell cultures

Primary neurons were prepared as previously described [4]. In brief, the hippocampus of the E18 mouse was homogenized and digested with 0.05% trypsin and cultured with neurobasal medium (Gibco, Grand Island, NY, USA) supplemented with 100 U/ml of penicillin, 100 μg/ml of streptomycin, 2% B27, and 0.5 mM L-glutamine. Neurons were seeded on poly-D-lysine-coated cell dishes for 7 days. Half of the culture media was changed once after 3.5 days. Mouse primary astrocytes and Neuro-2a neuroblastoma cells (ATCC, CCL-131) were cultured in MEM medium containing 10% fetal bovine serum, 2 mM L-glutamine, 100 U/ml of penicillin and 100 μg/ml of streptomycin. For the neuron-astrocyte co-culture experiment, Neuro-2a cells were seeded in the upper compartment of transwell inserts (1 μm pore membrane, Corning, NY, USA). Primary astrocytes were plated in the lower compartment of 12-transwell plates. Cells were grown at 37 °C in a 5% CO_2_ incubator. Primary hippocampal neurons were stimulated with a high glucose culture medium containing 100 mM glucose for 72 h. Neuro-2a cells were treated with 50 mM glucose for 48 h [4, 24]. SAHA (vorinostat, MCE, Monmouth, USA, 5 μM) and TSA (trichostatin A, MCE, 100 nM) were used as inhibitors of histone deacetylase. ACSS2-IN (Ac-CoA synthase inhibitor 1, MCE, 5 μM) and ACLY-IN (Bempedoic acid, MCE, 10 μM) were used as inhibitors of acetate-dependent acetyl-CoA synthetase 2 (ACSS2) and citrate-dependent ATP-citrate lyase (ACLY), respectively. KC7F2 (MCE, 40 μM) was used as a HIF-1 inhibitor. DCA (Sodium dichloroacetate, MCE, Monmouth, USA, 5 mM) was used as an inhibitor of pyruvate dehydrogenase kinases.

### Western blotting

Briefly, cultured cells or mouse hippocampus tissues were harvested and homogenized in a lysis buffer containing protease and phosphatase inhibitors. The protein concentration of each sample was quantified with a Pierce^TM^ BCA Protein Assay Kit (Thermo Fisher, MA, USA). Then, 30 μg proteins of each group were loaded on 12% SDS-PAGE gels and then transferred to PVDF membranes. After blocked with 5% milk or 3% bovine serum albumin, the membranes were incubated with primary antibodies against PDK1 (mouse, 1:500, Proteintech, Rosemount, IL, USA), β-actin (mouse, 1:4000, CST, Boston, USA), phospho-Ser^293^-PDH-E1α (rabbit, 1:500, Abcam, Cambridge, MA, USA), phospho-Ser^300^-PDH-E1α (rabbit, 1:1000, Proteintech), PDH-E1 (rabbit, 1:2000, Proteintech), H3K9ac (rabbit, 1:1000, CST), H3K14ac (rabbit, 1:1000, CST), H3K27ac (rabbit, 1:1000, CST), H3 (rabbit, 1:1000, CST), Bcl-2 (mouse, 1:500, Santa, CA, USA), Bax (rabbit, 1:1000, CST), Caspase-3 (rabbit, 1:1000, CST), active Caspase-3 (rabbit, 1:500, CST).

### RNA isolation and real-time quantitative PCR

TRIZOL reagent (Invitrogen, Carlsbad, USA) was used for RNA isolation. Total RNA concentration was measured by a spectrophotometer. The RevertAid^TM^ First Strand cDNA Synthesis Kit (Thermo Fisher) was applied to reverse transcription. Real-time PCR was performed with SYBR Green Realtime PCR Master Mix (TOYOBO, Osaka, Japan) and Bio-Rad CFX Connect detection system. *β-actin* gene is used as an internal control and the 2^−ΔΔCT^ method was used to calculate gene expression. The gene primer sequences were as follows: *Pdk1*, forward: 5′-AGG AAG TCC ATC TCA TCG-3′, reverse: 5′-AGC GTT CTC ATA GCC ATC-3′; *Pdk2*, forward: 5′-CAA TCA ACA CAC CCT CAT C-3′, reverse: 5′- CGT CTT TCA CCA CAT CAG-3′; *Pdk3*, forward: 5′- CGT CGC CAC TGT CTA TCA-3′, reverse: 5′-CAT GGT GTT AGC CAG TCG-3′; *Pdk4*, forward: 5′-TAC TCC ACT GCT CCA ACA C-3′, reverse: 5′-TGA TAG CGT CTG TCC CAT A-3′; *Hif-1α*, forward: 5′- CAT CAT CTC TCT GGA TTT TG-3′, reverse: 5′-GAA GAG GGA AAC ATT ACA TC-3′; *β-actin*, forward: 5′-CGT TGA CAT CCG TAA AGA CCT C-3′, reverse: 5′-CCA CCG ATC CAC ACA GAG TAC-3′.

### Immunofluorescence

Accordingly, mice were deeply anesthetized with barbiturate and perfused with saline and 10% phosphate-buffered formalin. Mouse brains were rapidly post-fixed in 10% formalin and embedded in paraffin. Then, 4-μm consecutive serial sections were obtained by using a Leica microtome. After deparaffinization, the sections were incubated in boiling antigen retrieval buffer (pH 6.0) for 8 min. Next, 3% hydrogen peroxide was used to block endogenous peroxidase activity. Cultured cells were fixed with 4% paraformaldehyde for 30 min. Cells and brain sections were blocked with 10% donkey serum and then incubated overnight at 4 °C with the following primary antibodies against HIF-1 (rabbit, 1:500, Servicebio, Wuhan, China), NeuN (mouse, 1:500, Millipore, MA, USA), PDK1 (rabbit, 1:500, Servicebio), MAP2 (mouse, 1:500, Proteintech). After washing three times with 0.1 M PBS, the cells and sections were incubated with secondary antibodies conjugated to Alexa Fluor 488 or Alexa Fluor 594 for 1 h at room temperature in dark conditions. The cells and brain sections were stained with 2 mg/ml DAPI solution for 10 min. Images were acquired on a VS120 Olympus fluorescence microscope. TUNEL staining was performed by the FITC green and TRITC Red Fluorescein in situ Apoptosis Detection Kit (Keygen Biotech, Nanjing, China).

### Chromatin immunoprecipitation assay

Chromatin immunoprecipitation (ChIP) was performed by using an EZ-ChIP kit (Millipore) according to the manufacturer’s protocol. In brief, cultured cells were fixed with 1% formaldehyde for 15 min at room temperature. The protein–DNA complexes were immunoprecipitated with anti-H3K9ac, anti-H3K14ac or control IgG overnight at 4 °C. The PCR primers of the *Pdk1* promoter in ChIP experiments were as follows: primer 1 (−0.5 kb), forward: 5′- ATC AGA GAG TTC CTC GTT C-3′, reverse: 5′- GAC TAG GAT TCA GGT AAG CT-3′; primer 2 (+0.5 kb), forward: 5′- TCA CTG GTG AGG AAG CTA A-3′, reverse: 5′- ACA CTA CCA TTC TGA GAC ACC-3′.

### Acetyl-CoA quantification

An acetyl-CoA assay kit (Solarbio Life Sciences, Beijing, China) was used to measure the cellular level of acetyl-CoA. A total of 5 × 10^6^ cells were collected and lysed in an extraction buffer for 30 min. After sonication, the cells were centrifuged at 8000 × *g* at 4 °C for 15 min and the supernatants were treated with acetyl-CoA assay buffer. The 340 nm absorbance values were measured at 20 s (A_20s_) and 80 s (A_80s_) by a microplate reader. The value (A_80S_-A_20S_) represented the relative level of acetyl-CoA.

### ROS production assay

To assess ROS production, the brain was quickly isolated and cut into 4-μm sections by using a freezing microtome. The brain sections were attached to chilled microscope slides carefully. For the in vitro experiment, cultured cells were plated on climbing slides. Both the cultured cells and the brain sections were incubated in saline containing 5 μM dihydroethidium (DHE, Beyotime, Shanghai, China) for 30 min at 37 °C in an incubator. MitoSOX Red (Yeasen, Shanghai, China) was used to detect superoxide in the mitochondria of neurons. The cultured cells were incubated in 3 μM MitoSOX Red (dissolved in HBSS) for 10 min at 37 °C. After incubation, the cells and brain sections were washed three times with 0.1 M PBS and photographed using a VS120 Olympus fluorescence microscope.

### Stereotaxic surgery and microinjection

Lenti-PDK1 or Lenti-GFP (1 × 10^8^ TU/ml, 1 μl per side, WZ Biosciences, Jinan, China) was infused bilaterally into the dorsal hippocampus 4 weeks before STZ injection by using a stereotaxic apparatus and a microsyringe connected with a microinjection pump (KD Scientific, Holliston, USA). The injection coordinates were as follows: anteroposterior, −1.7 mm; lateral, ±1.5 mm; dorsoventral, −2.3 mm.

Locomotor activity was tested in a 40 × 40 cm black box. Each mouse moves freely for 10 min in the box. The movements were recorded by a camera at the top and measured by a video tracking system.

The novel object recognition memory (ORM) and location-dependent ORM (OLM) tests were performed next as previously described [4, 25]. On day 1, mice were placed in the box for adaptation for 10 min. On day 2, mice were allowed to explore two identical objects (A_1_ and A_2_) for 5 min for familiarization. On day 3, mice were allowed to explore for 5 min. For ORM, the object A_2_ was changed into a new object B_1_ with a different shape. For OLM, the object A_2_ was placed in a new location. The exploration time was recorded when mice turned toward the object within 1 cm or when their noses were in contact with the object. The formula T_novel_/(T_novel_ + T_familiar_) was represented as the discrimination index.

The Morris water maze was performed by a circular tank (120 cm diameter, 40 cm height). In brief, the circular tank was divided into four identical quadrants, and an escape platform (6 cm diameter) was placed in the target quadrant under the water. Mice were subjected to five consecutive days of training and four times each day. Mice were allowed to find the escape platform within 60 s and stay on the platform for 30 s each time. On day 6, the platform was removed and mice were recorded to explore 60 s (probe trial). The time spent in each quadrant and the number of platform crossings were analyzed with a camera and a video tracking system (Clever Sys Inc., Reston, USA).

### Statistical analysis

Data analysis was carried out with the SPSS statistical program, version 19.0. The results are presented as the mean ± SEM. Data differences between the two groups were analyzed by Student’s *t*-test, one-way or two-way ANOVA followed by the LSD or Dunnett’s T3 post hoc test. Results with *p* < 0.05 were considered to be statistically significant. For in vitro experiments, data represent the mean of at least three independent experiments. For animal behavior tests, sample sizes are at least 6 per group. When possible, investigators were blinded during group allocation, behavior tests and raw data statistics.

## Results

### PDK1 is decreased in neurons of the hippocampus in diabetic mice and increased in high glucose-treated primary neurons

**Fig. 1:**
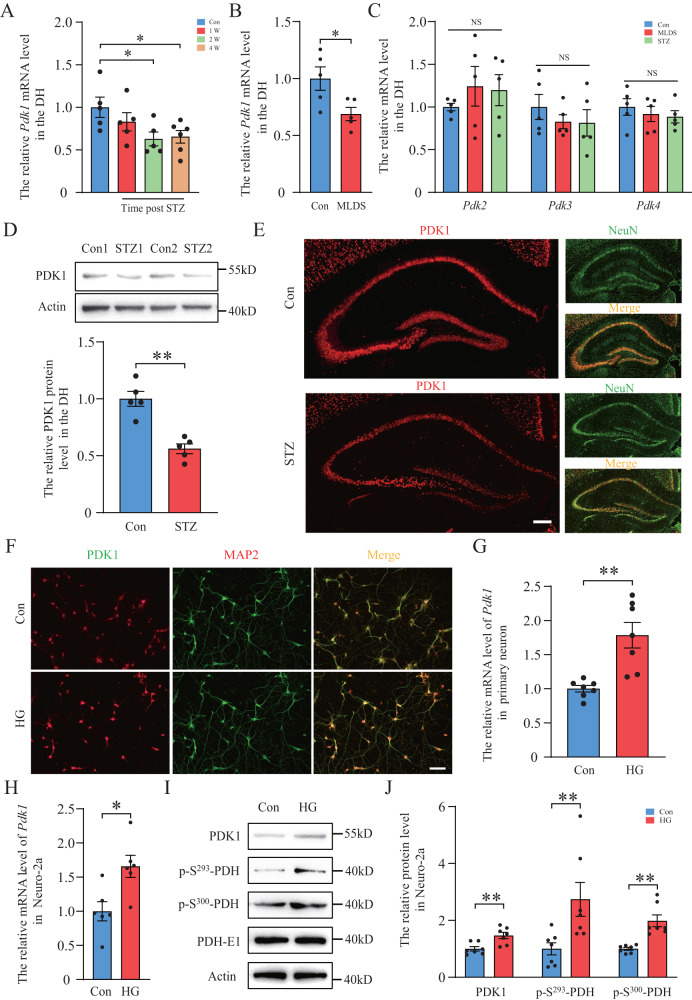
PDK1 expression in the dorsal hippocampus of STZ-induced diabetic mice and high glucose-stimulated neuronal cells. **A** The expression of *Pdk1* mRNA in the dorsal hippocampus (DH) at 1, 2 and 4 w following a single STZ injection (*n* = 5–6 per group). **B** The expression of *Pdk1* mRNA in the DH 4 w after multiple injections of low doses of STZ (MLDS) (*n* = 5 per group). **C** The expression of *Pdk* isoform mRNAs in the DH 4 w after STZ injection (*n* = 5 per group). **D** Western blot analysis of PDK1 in the DH of each group (*n* = 5 per group). **E** Immunofluorescent staining of PDK1 and neuronal marker NeuN in the DH in the control group and STZ-treated group. Scale bar = 200 μm. **F** Immunofluorescent staining of PDK1 and neuronal marker MAP2 in primary neurons in the control group and high glucose-treated group. Scale bar = 50 μm. **G**, **H** The expression of *Pdk1* mRNA in each group in primary neuron and Neuro-2a (*n* = 6–7 per group). **I**, **J** Western blot analysis of PDK1, p-S^293^-PDH, p-S^300^-PDH and PDH-E1 in each group (*n* = 7 per group). *p < 0.05, **p < 0.01, *t*-test or one-way ANOVA. Data represent the mean of at least three independent experiments.

Previous studies have proved that neuronal injury in the hippocampus may lead to memory loss in diabetic mice [26, 27]. We examined the expression of PDK1 in the dorsal hippocampus (DH) of diabetic mice. Two diabetic models were used in this experiment: a single high dose of streptozotocin (STZ) injection or multiple low doses of STZ (MLDS) injection for 5 consecutive days. Quantitative PCR results showed that *Pdk1* was significantly reduced after 2 weeks and 4 weeks after a single injection of STZ (Fig. 1A). *Pdk1* was also decreased in the DH of MLDS group mice 4 weeks after injection (Fig. 1B). But the mRNA levels of *Pdk2, Pdk3* and *Pdk4* had no obvious change in diabetic mice compared with the control group (Fig. 1C). By using western blot and immunofluorescent staining, we found that PDK1 was mainly expressed in neurons and obviously decreased in the DH of diabetic mice (Fig. 1D, E). We also examined the expression of PDK1 in high glucose (HG)-treated primary neuron and Neuro-2a cell line. Stimulation of HG significantly increased the expression of PDK1 in both primary neurons and Neuro-2a cells (Fig. 1F–J). Similarly, the phosphorylation levels of PDH (p-S^293^-PDH and p-S^300^-PDH) were enhanced after HG treatment (Fig. 1I, J). The opposite results of PDK1 expression in diabetic mice and HG-treated neurons encouraged us to focus more attention on the transcriptional regulation mechanism of the *Pdk1* gene.

### PDK1 expression is regulated by acetyl-coA-induced histone acetylation in HG-treated neurons

**Fig. 2:**
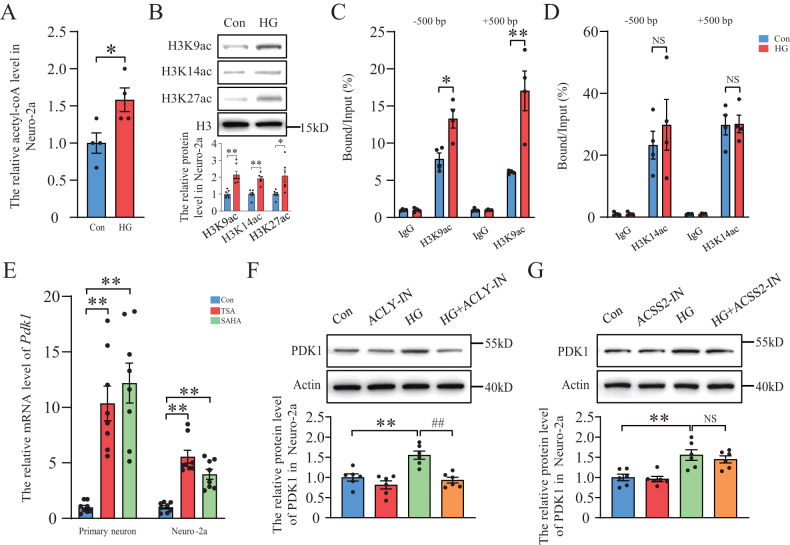
PDK1 expression is mediated by acetyl-coA-induced histone acetylation in high glucose-stimulated neurons. **A** Quantification of acetyl-coA level in high glucose-treated Neuro-2a (*n* = 4 per group). **B** Western blot analysis of H3 and histone acetylation of H3K9, H3K14 and H3K27 after high glucose stimulation (*n* = 6–7 per group). **C**, **D** Chromatin immunoprecipitation (ChIP) analysis of the histone acetylation levels of H3K9 and H3K14 in the promoter region of the *Pdk1* gene in each group (*n* = 4 per group). DNA from each ChIP sample was normalized by the corresponding input sample. **E** The expression of *Pdk1* mRNA in HDAC inhibitor TSA and SAHA-treated primary neuron and Neuro-2a (*n* = 8 per group). **F**, **G** Western blot analysis of PDK1 after stimulation of ACLY-IN, ACSS2-IN and high glucose in Neuro-2a (*n* = 6 per group). *p < 0.05, **p < 0.01 versus the control group, ^##^*p* < 0.01 versus the high glucose group, two-way ANOVA.

It has been reported that HG can increase acetyl-coA-induced global histone hyperacetylation and gene expression in mesangial cells [28–30]. Therefore, we investigated the effect of acetyl-coA-induced histone acetylation on PDK1 expression. First, we found that the intracellular level of acetyl-coA in Neuro-2a was increased after HG treatment (Fig. 2A). Next, our results showed that the levels of H3K9, H3K14 and H3K27 acetylation were significantly increased in HG-treated group (Fig. 2B). Our ChIP data showed that the level of H3K9 acetylation at the promoters of the *Pdk1* gene was significantly increased after HG stimulation while the level of H3K14 acetylation was not changed (Fig. 2C, D). Furthermore, two histone deacetylase inhibitors (HDACi), TSA and SAHA, were used to enhance the level of histone acetylation in neurons. We found that TSA and SAHA largely increased the mRNA level of *Pdk1* in both primary neuron and Neuro-2A (Fig. 2E). These data suggested that HG-induced histone acetylation promotes the expression of PDK1 in neurons. Acetate-dependent acetyl-CoA synthetase 2 (ACSS2) and citrate-dependent ATP-citrate lyase (ACLY) are two principal enzymes that generate acetyl-CoA for histone acetylation. ACLY converts glucose-derived citrate into acetyl-CoA while ACSS2 relies on acetate metabolism for histone acetylation. Two inhibitors of ACSS2 and ACLY, ACSS2 inhibitor 1 (ACSS2-IN) and bempedoic acid (ACLY-IN) respectively, were used in this experiment. Western blot data showed that ACLY-IN, but not ACSS2-IN, completely abolished the HG-induced increase of PDK1 expression in Neuro-2a (Fig. 2F, G). Therefore, the above data indicated that HG-increased PDK1 expression may rely on acetyl-coA-induced histone acetylation.

### HIF-1 participates in HG-induced transcriptional activation of *Pdk1* and is reduced in neurons of the hippocampus in diabetic mice

**Fig. 3:**
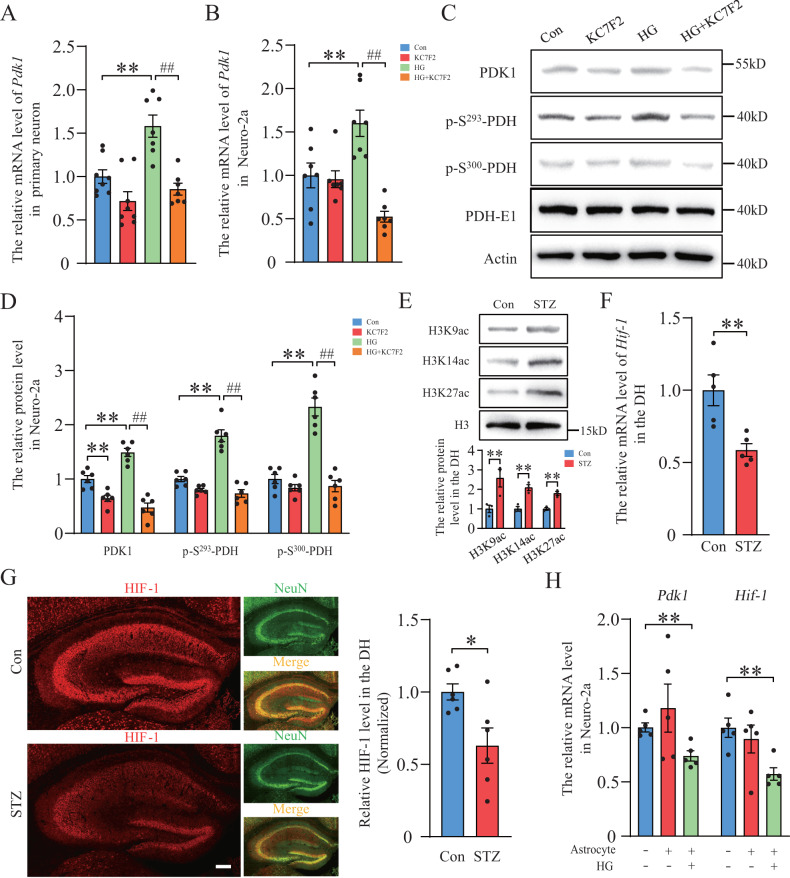
HIF-1 directly regulates PDK1 expression under high glucose condition and is reduced in the hippocampus of diabetic mice. **A**, **B** The expression of *Pdk1* mRNA in high glucose and HIF-1 inhibitor KC7F2-treated primary neuron and Neuro-2a (*n* = 7–8 per group). **C**, **D** Western blot analysis of PDK1, p-S^293^-PDH, p-S^300^-PDH and PDH-E1 in each group (*n* = 6 per group). **p < 0.01 versus the control group, ^##^*p* < 0.01 versus the high glucose group, two-way ANOVA. **E** Western blot analysis of H3 and histone acetylation of H3K9, H3K14 and H3K27 in the hippocampus of diabetic mice (*n* = 3 per group). **F** The expression of *Hif-1* mRNA in the hippocampus of diabetic mice (*n* = 5 per group). **G** Immunofluorescent staining of HIF-1 and NeuN, and quantification of HIF-1 expression in the hippocampus in the control group and STZ-treated group (*n* = 6 per group). Scale bar = 200 μm. **H** The expression of *Pdk1* and *Hif-1* mRNA in Neuro-2a after co-culture with primary astrocyte under high glucose condition for 48 h (*n* = 5 per group). *p < 0.05, **p < 0.01, *t*-test or one-way ANOVA.

As previously described, PDK1 is an important HIF-1-target gene in hypoxic B lymphocyte cell line [23]. In this study, HG-increased the mRNA level of *Pdk1* was reversed in HIF-1 inhibitor KC7F2-treated primary neuron and Neuro-2a (Fig. 3A, B). Western blot data also showed that KC7F2 prevented the protein level of PDK1 and phosphorylation of PDH that were enhanced in the HG treatment group (Fig. 3C, D). This suggested that both acetyl-coA-induced histone acetylation and HIF-1 are necessary factors to regulate the expression of the *Pdk1* gene in HG condition. To investigate the possible mechanism of diabetic hyperglycemia-impaired PDK1 expression in neurons of the hippocampus, we examined whether the level of histone acetylation and the expression of HIF-1 were changed in vivo. Consistent with in vitro experiments, the acetylation levels of H3K9, H3K14 and H3K27 were highly increased in the DH of diabetic mice (Fig. 3E). However, STZ injection decreased the expression of HIF-1 in neurons of the DH (Fig. 3F, G). This finding may explain the opposite expression of PDK1 in HG-treated neurons and diabetic mice. Although the increase of histone acetylation was not impaired, inhibition of HIF-1 signaling might destroy PDK1 expression and the self-protective effect of neurons. In the CNS, astrocyte plays a key role in energy metabolism and maintaining neuronal activity. Therefore, we investigated the effect of astrocyte on the expression of PDK1 and HIF-1 in neurons. By using a transwell stereo culture, our data showed that co-culture with astrocyte under high glucose conditions significantly decreased the mRNA levels of *Pdk1* and *Hif-1* in Neuro-2a (Fig. 3H).

### Overexpression of PDK1 alleviated HG-induced ROS production and neuronal apoptosis

**Fig. 4:**
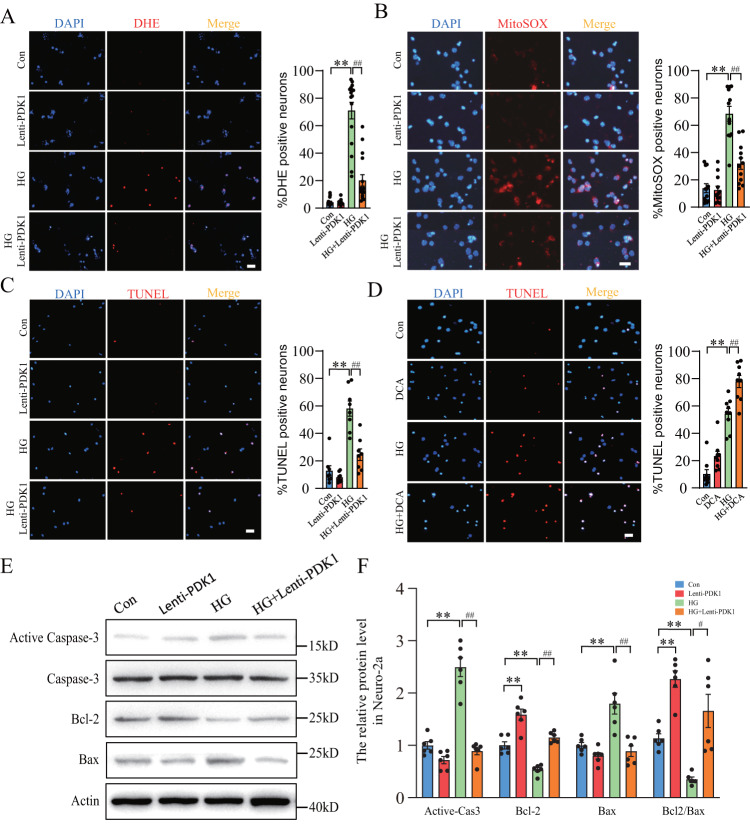
Overexpression of PDK1 decreased high glucose-induced ROS production and neuronal apoptosis. **A** DHE staining and quantification of DHE-positive primary neurons in each group (*n* = 16 per group). **B** MitoSOX staining and quantification of mitoSOX-positive primary neurons in each group (*n* = 12 per group). **C**, **D** TUNEL staining and quantification of TUNEL-positive primary neurons in each group (*n* = 8–9 per group). Scale bar = 50 μm. **E**, **F** Western blot analysis of Bcl-2, Bax, active Caspase-3 and total Caspase-3 in each group (*n* = 6 per group). **p < 0.01 vs. the control group; ^#^*p* < 0.05, ^##^*p* < 0.01 vs. the high glucose group, two-way ANOVA.

To investigate the effect of PDK1 on HG-induced oxidative stress and neuronal apoptosis, overexpression of PDK1 by lentivirus (Lenti-PDK1) was used in primary neurons and Neuro-2a. Reactive oxygen species (ROS) and neuronal apoptosis were measured by dihydroethidium (DHE), mitoSOX and TUNEL staining, respectively. As shown in Fig. 4A, B, HG increased the number of DHE-positive and MitoSOX-positive neurons. Compared with the HG-treated group, overexpression of PDK1 significantly prevented the increased number of DHE-positive and MitoSOX-positive neurons. This suggested that inhibition of pyruvate metabolism into acetyl-coA might alleviate the overload of mitochondrial metabolism and reduce the release of ROS by the mitochondrial electron transfer chain complex. TUNEL staining showed that Lenti-PDK1 significantly reversed HG-induced the increase of TUNEL-positive neurons (Fig. 4C). As an inhibitor of pyruvate dehydrogenase kinases, DCA further aggravated HG-induced neuronal apoptosis (Fig. 4D). We also examined the protein level of active Caspase-3, Bcl-2 and Bax by western blot. Our data displayed a significant increase of active Caspase-3 under HG condition in Neuro-2a, which was totally abolished in the Lenti-PDK1-treated group (Fig. 4E, F). In addition, overexpression of PDK1 prevented the increase of Bax and the decrease of Bcl-2 induced by HG (Fig. 4E, F). Our data demonstrated that PDK1 alleviated HG-induced oxidative stress and neuronal apoptosis.

### PDK1 reduced STZ-induced ROS production in the hippocampus and rescued memory loss in mice

**Fig. 5:**
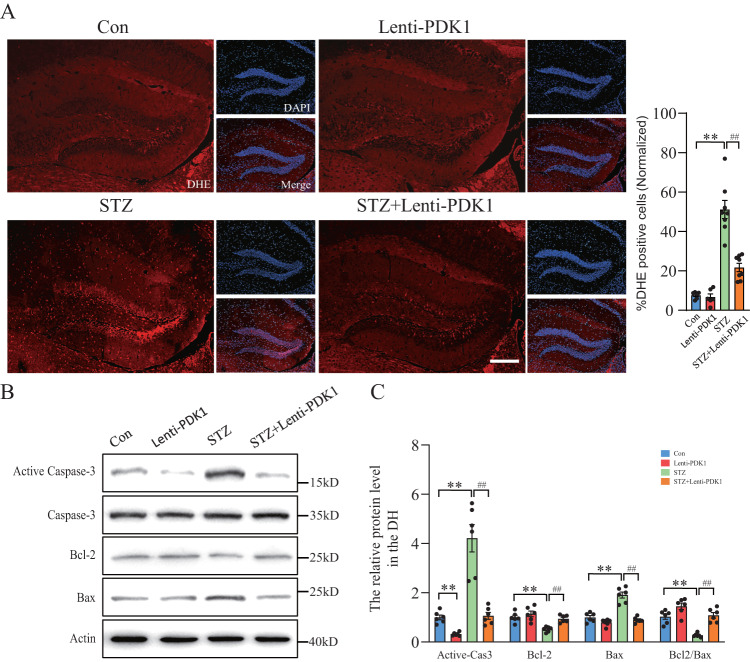
PDK1 reduced STZ-induced ROS production and neuronal apoptosis in the hippocampus of mice. **A** DHE staining and quantification of DHE-positive cells in the hippocampus of Lenti-PDK1-injected diabetic mice (*n* = 6–8 per group). Scale bar = 200 μm. **B**, **C** Western blot analysis of Bcl-2, Bax, active Caspase-3 and total Caspase-3 in each group (*n* = 6 per group). **p < 0.01 vs. the control group, ^##^*p* < 0.01 vs. the STZ injection group, two-way ANOVA.

Next, we investigated the role of PDK1 in the hippocampus of diabetic mice. ROS production in the DH was detected by DHE fluorescence staining as previously described [31]. STZ-treated mice showed an increase of DHE-positive cells in the DH (Fig. 5A). Interestingly, injection of Lenti-PDK1 significantly reduced STZ-induced increase of DHE-positive cells, which suggested that overexpression of PDK1 in the DH limited STZ-induced release of ROS. Western blot data also displayed that overexpression of PDK1 reversed STZ-induced increases of active Caspase-3 and Bax, and decrease of Bcl-2 (Fig. 5B, C). Our data proved that reprogramming of pyruvate metabolism in mitochondria by PDK1 may alleviate diabetic hyperglycemia-induced mitochondrial production of ROS, and further rescued neuronal apoptosis in the hippocampus.

**Fig. 6:**
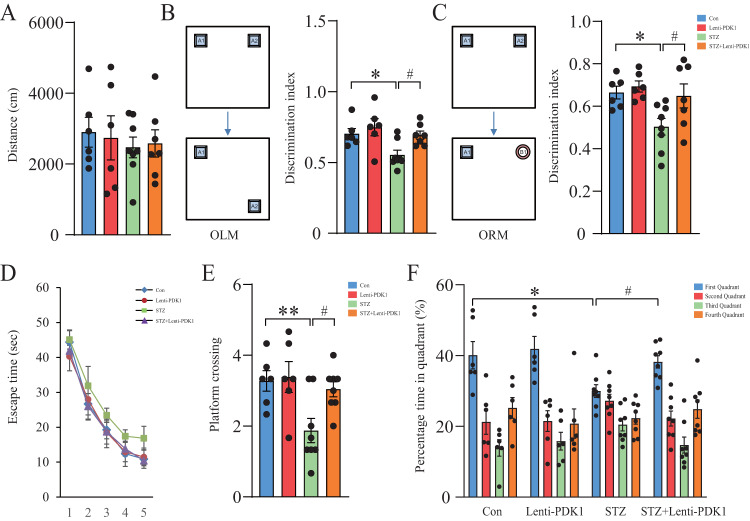
Hippocampal injection of Lenti-PDK1 alleviated STZ-induced memory loss in mice. **A** Locomotor activity was quantified 4 weeks after microinjection of Lenti-GFP or Lenti-PDK1 into the dorsal hippocampus of mice (*n* = 6–8 per group). **B** Schematic representation of the location-dependent object recognition memory (ORM) test (Left). Quantification of discrimination index in each group (*n* = 6–8 per group; Right). **C** Schematic representation of the novel ORM test (Left). Quantification of discrimination index in each group (*n* = 6–8 per group; Right). **D** The escaped latency to find the hidden platform in the consecutive five training days in the Morris water maze test. **E** The times of platform crossing in the target quadrant in the probe trial (*n* = 6–8 per group). **F** The time spent in the target quadrant in the probe test (*n* = 6–8 per group). *p < 0.05, **p < 0.01 vs. the control group; ^#^*p* < 0.05 vs. the STZ injection group, two-way ANOVA.

As previously described, hippocampal neuronal apoptosis can lead to cognitive impairment in diabetic mice [4, 27]. Therefore, we investigated the effect of PDK1 on STZ-induced memory loss by using the novel object recognition memory (ORM) and the Morris water maze tests. As shown in Fig. 6A, STZ and injection of Lenti-PDK1 showed no obvious change in mouse locomotor activity. In ORM and OLM tests, STZ mice exhibited decreased discrimination ability compared with the control group, which was significantly rescued by overexpression of PDK1 in the hippocampus of mice (Fig. 6B, C). Similarly, in the water maze test, STZ mice showed increased escape latency in the hidden platform trials, decreased numbers of platform crossing and time in the target quadrant (Fig. 6D–F). Overexpression of PDK1 in the hippocampus significantly reversed STZ-induced memory loss in the water maze test. Thus, all the above results suggested that PDK1 in the hippocampus has the ability to prevent hyperglycemia-induced neuronal apoptosis and improve cognitive impairment in diabetic mice.

## Discussion

Hyperglycemia brings extensive and long-lasting neurological disorders in diabetes [2, 6]. We have focused on reprogramming hyperglycemia-induced aberrant glucose metabolism to explore effective and available treatment for many years [4]. The PDK-lactic acid axis is a crucial regulator of glucose metabolism and orchestrates the destination of glucose according to different energy supply status [11]. In this study, we reveal a novel neuroprotective effect of PDK1 in diabetic neuropathy. We initially found that PDK1 was decreased in neurons of the hippocampus in diabetic mice and increased in HG-stimulated primary neurons. Second, acetyl-coA, a central metabolite and second messenger, enhanced PDK1 expression in an epigenetic manner in HG-treated neurons. This provided an important negative feedback regulation to inhibit the activity of PDH and prevent HG environment-triggered mitochondrial metabolic overload. Third, HG-enhanced PDK1 expression also relies on transcriptional activation of HIF-1. The decrease of HIF-1 in the hippocampus of diabetic mice may explain the impaired expression of PDK1 in vivo. Finally, overexpression of PDK1 reduced HG-induced ROS production, and ameliorated neuronal apoptosis and memory loss in diabetic mice.

PDK1 is regarded as a glycolysis gatekeeper and plays key roles in tumor growth and metastasis, macrophage polarization, and right ventricular fibrosis [32–34]. PDK1 is highly expressed in neurons of the CNS [14]. An increase in PDK1 expression is observed in Aβ-resistant nerve cells. Overexpression of PDK1 confers resistance to Aβ and other neurotoxins in vitro, which suggests that PDK1 may play a protective effect in Alzheimer’s disease [15, 16]. A new study has reported that astrocytic PDK2 is increased in the hypothalamus of diabetic mice, and involved in hypothalamic inflammation and disrupting feeding behavior through its metabolic regulation [12]. However, the effect of PDK1 in diabetic hyperglycemia-induced neurological disease is ignored. Our data showed a significant decrease of PDK1 in neurons of the hippocampus in mice models of diabetes. However, we observed that PDK1 is significantly increased in HG-treated primary neurons and Neuro-2a. The activity of PDH is also enhanced after HG treatment. This opposite result provoked us into thinking the possible explanation in the following work.

Acetyl-coA is a central metabolite and acts as a second messenger, which can influence multiple metabolic enzymes activity by its cellular concentrations and direct gene expression via acetylation-induced epigenetic modification [35]. It is reported that acetyl-coA-mediated histone acetylation participates in neural stem cell differentiation, hippocampal memory formation and glioblastoma survival [36–38]. We found that HG increases the level of acetyl-coA in cultured neurons. The levels of histone acetylation are significantly increased in HG-treated neurons. Therefore, we investigated the effect of acetyl-coA-regulated histone acetylation on PDK1 expression. ChIP data showed that the level of H3K9 acetylation at the promoter of the *Pdk1* gene was significantly enhanced after HG stimulation. Stimulation with HDACi, TSA and SAHA, largely increased the expression of PDK1. These results suggested that HG induces PDK1 expression via upregulating the activity of the *Pdk1* promoter. As previously described, ACSS2 and ACLY are two principal enzymes that generate acetyl-CoA for histone acetylation [37]. ACLY exists in both mitochondria and nucleus. Acetyl-CoA can be transferred from mitochondria to the cytosol and nucleus by means of the export of citrate and the cleavage activity of ACLY. ACLY has been reported to directly regulate histone acetylation levels in diverse mammalian cell types [30, 39]. In this study, we found that inhibition of ACLY completely abolished the HG-induced increase of PDK1 protein in Neuro-2a. But ACSS2-IN has no effect on PDK1 expression under HG condition. We indicated that HG-enhanced PDK1 expression may rely on acetyl-coA-induced histone acetylation in an ACLY-dependent manner. Therefore, a possible self-protective model of neuron is proposed, which states that excessive acetyl-coA can be generated from mitochondrial aerobic metabolism, enhance PDK1 expression in an epigenetic manner and further avoid HG-triggered mitochondrial metabolic overload by inhibition of PDH activity.

Transcriptional activation of genes encoding glucose transporters and glycolytic enzymes by HIF-1 is considered critical for metabolic adaptation to hypoxia, which can orchestrate increased anaerobic glycolysis flux through the conversion of glucose to lactate [23, 40]. Under hypoxic circumstance, HIF-1 directly activates *Pdk1* expression and suppresses metabolism through the tricarboxylic acid cycle (TCA) in the B lymphocyte cell line [23]. Therefore, we are interested in the effect of HIF-1 in PDK1 expression under HG condition. Data showed that HIF-1 inhibitor blocked HG-increased PDK1 expression and phosphorylation of PDH in primary neuron and Neuro-2a. We indicated that both acetyl-coA-induced histone acetylation and HIF-1 signaling are necessary for transcription regulation of PDK1 expression. In addition, we examined the level of histone acetylation and HIF-1 expression to fully explain the decrease of PDK1 in the hippocampus of STZ mice. Injection of STZ largely increased acetylation levels of H3K9, H3K14 and H3K27 in the hippocampus. However, the expression of HIF-1 in neurons was reduced in diabetic mice. In accordance with this, a recent study reported that HIF-1 was repressed in a prolyl-hydroxylase-dependent manner in hypoxic renal mIMCD-3 cells and in the kidneys of animals with diabetes [40]. Therefore, we demonstrated that although the levels of histone acetylation were significantly enhanced, inhibition of HIF-1 signaling might decrease PDK1 expression and impair the self-protective effect of neurons in the hippocampus of diabetic mice.

In this study, there may be several reasons for the decreases of HIF-1 and PDK1 in the hippocampus of diabetic mice. First, the effect of astrocyte and microglia in the hippocampus cannot be neglected. Under hyperglycemia conditions, astrocyte-neuron metabolic cooperation may be disrupted [41–43]. Astrocyte and microglia-mediated neuroinflammation may also be increased [12, 44]. Our data has shown that co-culture with astrocyte under HG conditions decreased the expression of *Pdk1* and *Hif-1* in Neuro-2a cells. Second, evidence revealed that under diabetic and hypoxia conditions, HIF-1 is inhibited by high glucose levels through a prolyl-hydroxylase-dependent mechanism in the kidneys [40]. Hyperglycemia may result in excessive consumption of oxygen in neurons and cause a state of local temporary hypoxia in the brain, which may explain the decrease of HIF-1 in the hippocampus of diabetic mouse. Third, under hyperglycemia condition, a lot of toxic metabolites accumulate in the blood and are transported into the brain regions. These effects on neuronal activity and survival cannot be ignored. Therefore, much more research should be investigated in the future to explain the decrease of HIF-1 and PDK1 in the hippocampus of diabetic mice.

Although PDK1 is highly expressed in neurons, its roles in the physiology and pathology process of the CNS are not well-established. It is found that PDK1 is elevated in Aβ resistant nerve cell lines and decreased in post-mortem brains of AD patients [15, 17]. Overexpression of PDK1 confers resistance to Aβ and reduces ROS production. PDK1-directed glucose metabolism programming showed a neuronal protective effect in AD, even though there is still a lack of sufficient evidence in vivo. In this work, we found that upregulation of PDK1 alleviated HG-induced ROS production and neuronal apoptosis in primary neurons and the Neuro-2a cell line. More importantly, DHE fluorescence staining displayed a significant decrease of DHE-positive cells in the hippocampus of Lenti-PDK1-injected diabetic mice, suggesting that PDK1 might prevent the mitochondrial TCA cycle and reduce ROS production in STZ mice. TUNEL staining and western blot data also revealed that overexpression of PDK1 reversed diabetic hyperglycemia-induced neuronal apoptosis in the hippocampus. Increased generation of ROS by the mitochondrial electron transfer chain is a major cause of hyperglycemia-induced cellular injury. PDK1-regulated glucose metabolism provides a valid and effective strategy to ameliorate hyperglycemia-induced oxidative stress. Finally, our ORM and Morris water maze behavior tests displayed that PDK1 in the hippocampus attenuated hyperglycemia-induced memory loss in mice model of diabetes.

In conclusion, our findings revealed a novel self-protective mechanism of neurons that PDK1 is directly upregulated by acetyl-coA-mediated histone acetylation and prevents HG-triggered mitochondrial metabolic overload and oxidative stress through inhibition of PDH activity. However, the decrease of HIF-1 in the hippocampus impaired PDK1 expression and neuronal protective effect in diabetic mice. The promising and available effect of PDK1 in hyperglycemia-induced neuronal apoptosis and memory loss will identify the PDK1-lactic acid axis as a therapeutic target for the treatment of diabetic neuropathy.

### Supplementary information

Reproducibility Checklist

Western blot original images

## Data Availability

All data generated or analyzed during this study are available from the corresponding author upon reasonable request.
