# Supplementary material for: Pyruvate dehydrogenase kinase 1 protects against neuronal injury and memory loss in mouse models of diabetes
Source: Cell Death Dis. 2023 Nov 7;14(11):722. doi: 10.1038/s41419-023-06249-2 (PMC10630521; doi:10.1038/s41419-023-06249-2)
Supplement: Supplementary file 2 — Western blot original images [file 41419_2023_6249_MOESM2_ESM.pptx]

## Slide 1
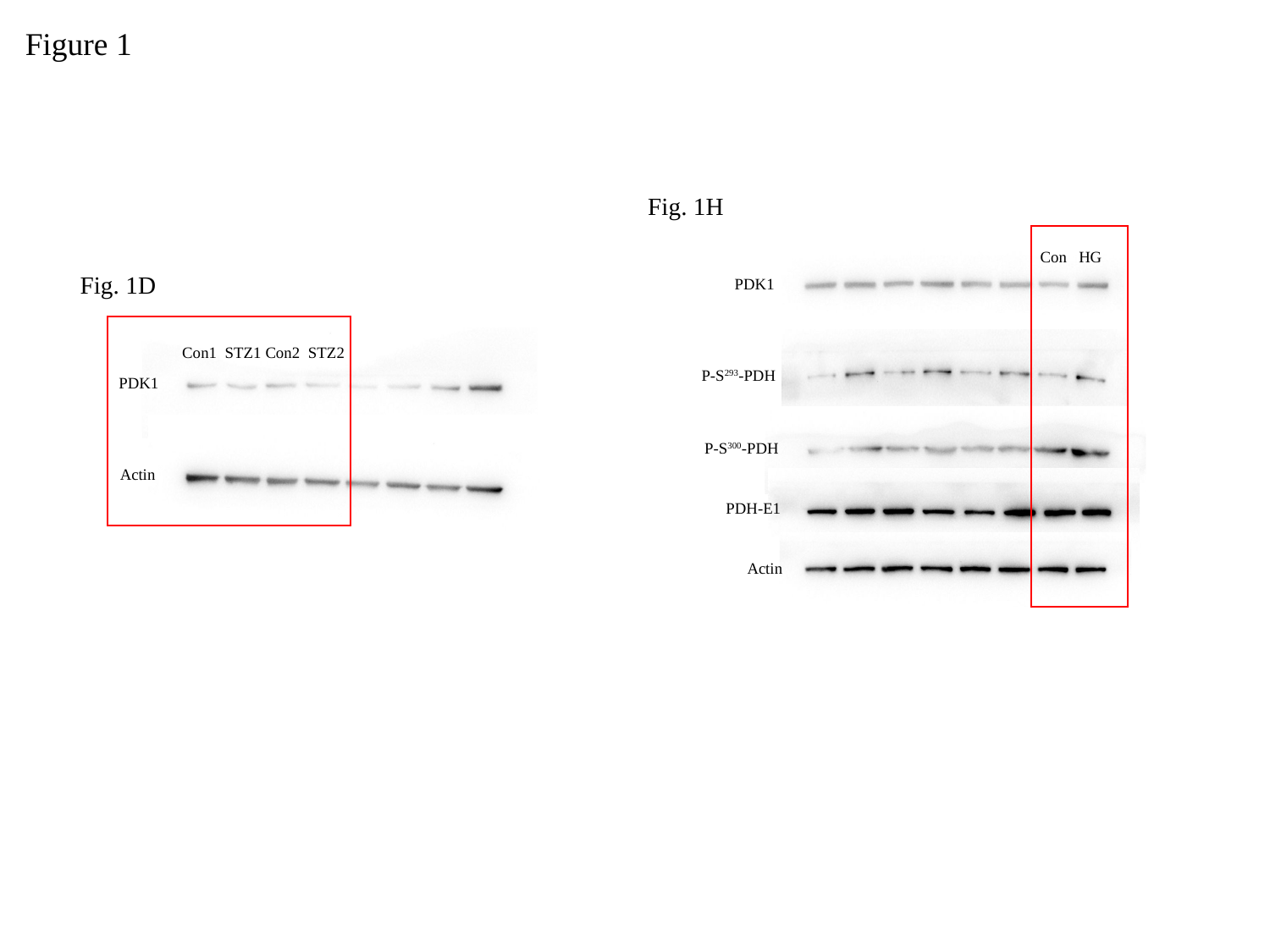

Figure 1
Fig. 1H
Con HG
PDK1
P-S293-PDH
P-S300-PDH
PDH-E1
Actin
Fig. 1D
Con1 STZ1 Con2 STZ2
PDK1
Actin

## Slide 2
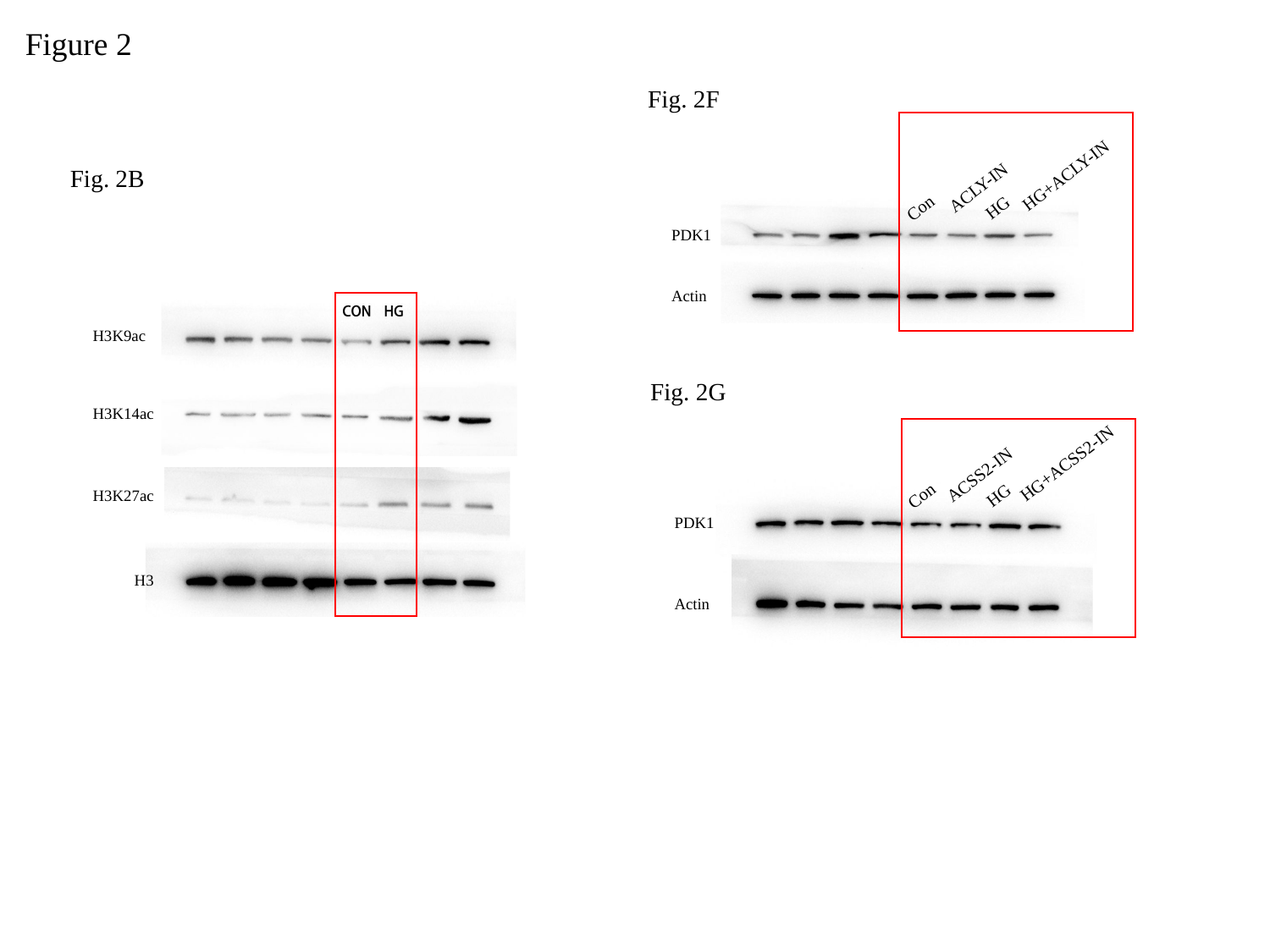

Figure 2
Fig. 2F
Fig. 2B
HG+ACLY-IN
ACLY-IN
HG
Con
PDK1
Actin
H3K9ac
H3K14ac
H3K27ac
H3
Fig. 2G
HG+ACSS2-IN
ACSS2-IN
HG
Con
PDK1
Actin

## Slide 3
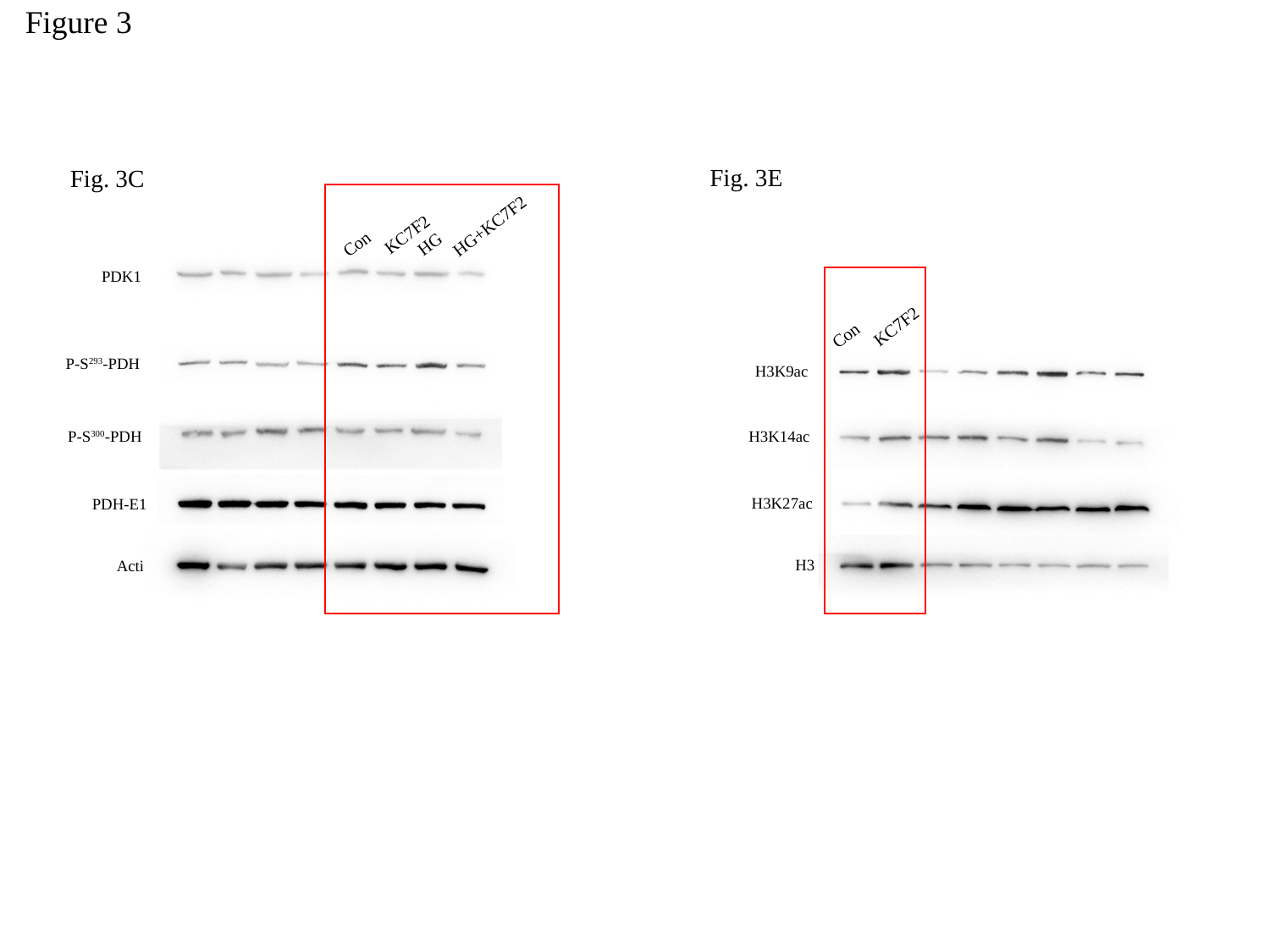

Figure 3
Fig. 3E
Fig. 3C
HG+KC7F2
KC7F2
HG
Con
PDK1
KC7F2
Con
H3K9ac
H3K14ac
H3K27ac
H3
P-S293-PDH
P-S300-PDH
PDH-E1
Actin

## Slide 4
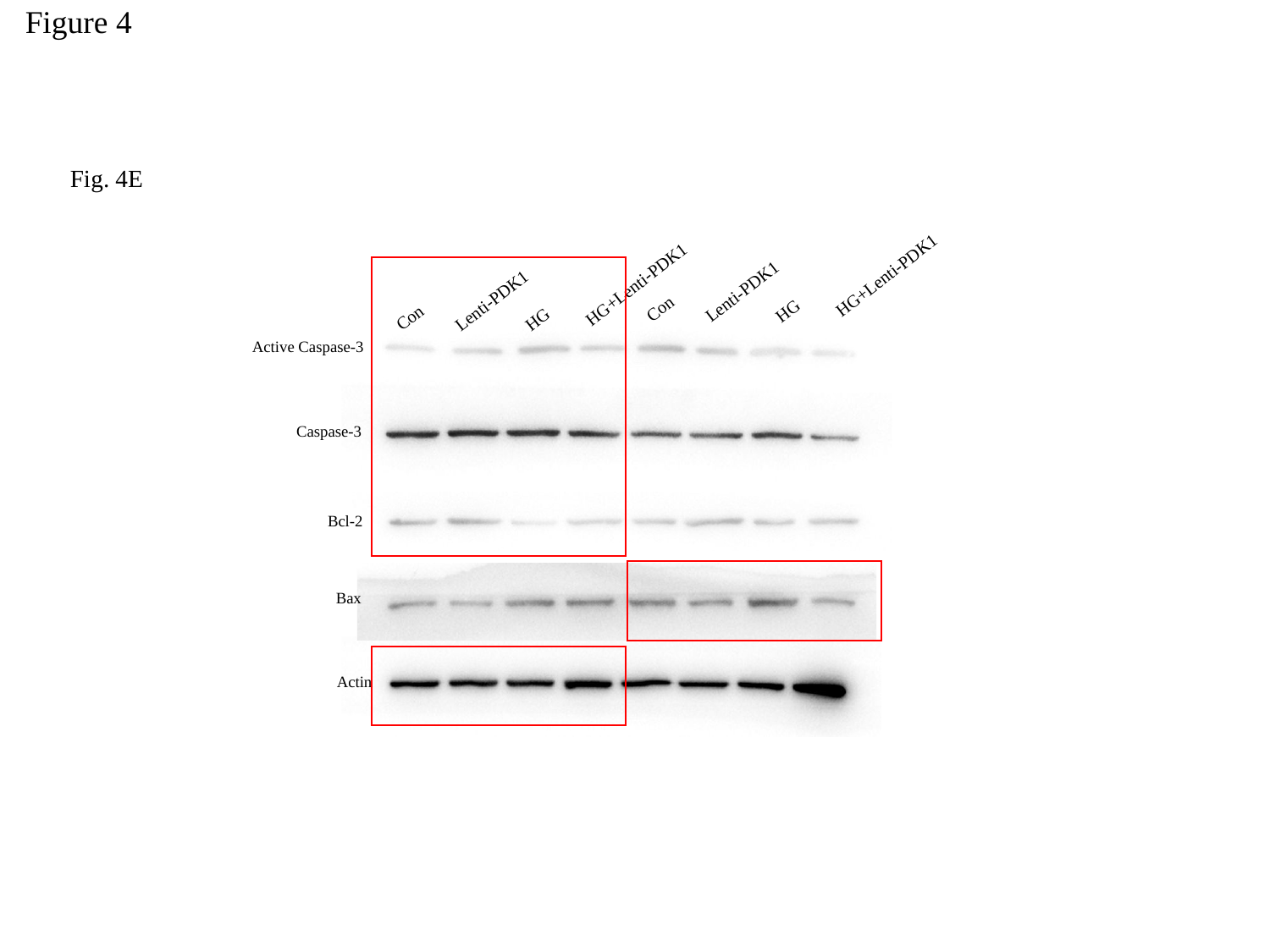

Figure 4
Fig. 4E
HG+Lenti-PDK1
HG+Lenti-PDK1
Lenti-PDK1
Lenti-PDK1
Con
HG
Con
HG
Active Caspase-3
Caspase-3
Bcl-2
Bax
Actin

## Slide 5
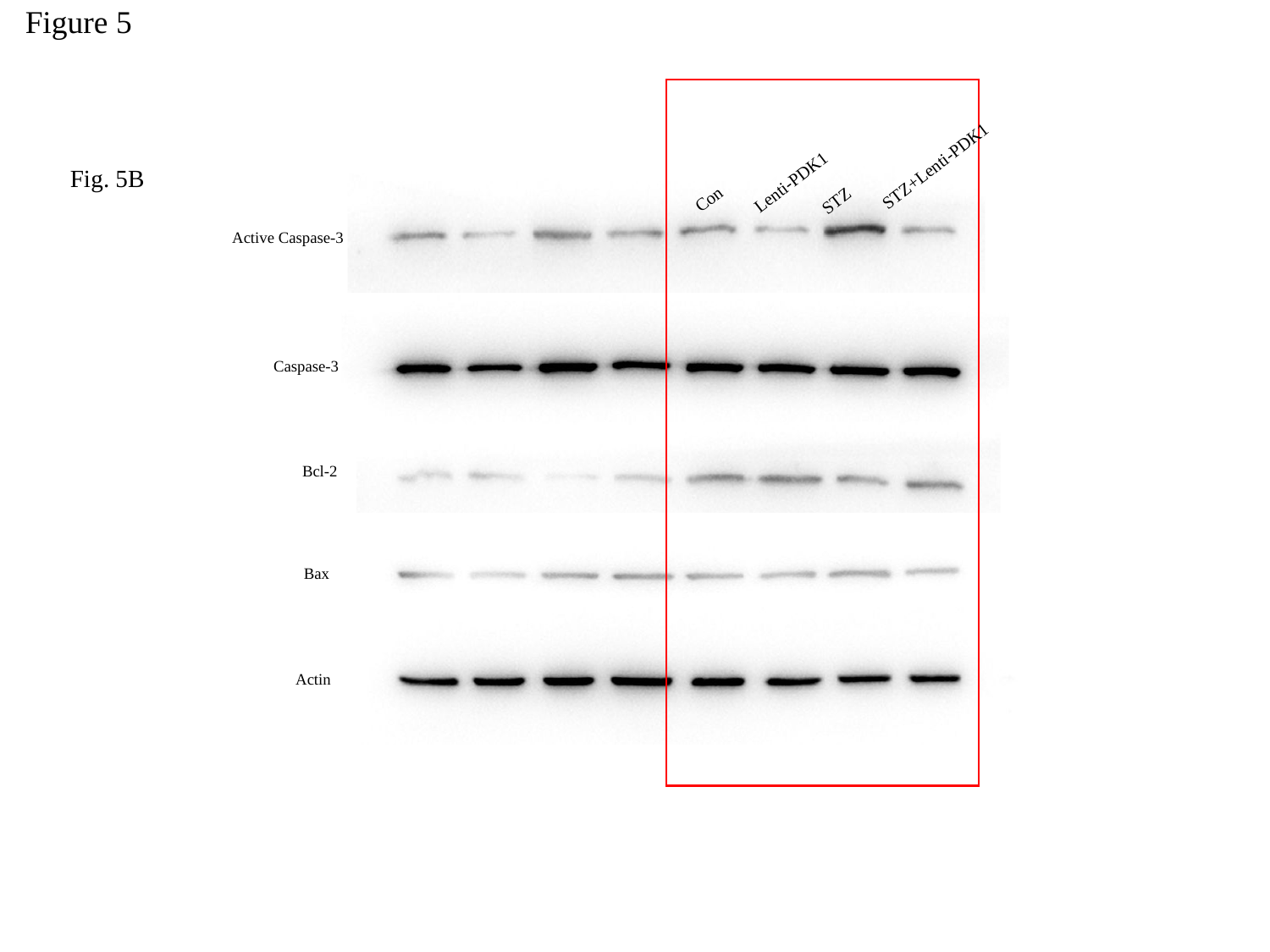

Figure 5
STZ+Lenti-PDK1
Fig. 5B
Lenti-PDK1
Con
STZ
Active Caspase-3
Caspase-3
Bcl-2
Bax
Actin
